# Astrocytes Control Neuronal Excitability in the Nucleus Accumbens

**DOI:** 10.1100/tsw.2007.195

**Published:** 2007-11-02

**Authors:** Tommaso Fellin, Marcello D'Ascenzo, Philip G. Haydon

**Affiliations:** ^1^ Department of Neuroscience, University of Pennsylvania School of Medicine, Philadelphia, PA 19104, USA; ^2^ Institute of Human Physiology, Catholic University, Largo Francesco Vito 1, 00168 Roma, Italy

**Keywords:** glia, astrocytes, glutamate release, gliotransmission, glutamate, metabotropic glutamate receptors, neuronal excitability, medium spiny neurons, drug addiction

## Abstract

Though accumulating evidence shows that the metabotropic glutamate receptor 5 (mGluR5) mediates some of the actions of extracellular glutamate after cocaine use, the cellular events underlying this action are poorly understood. In this review, we will discuss recent results showing that mGluR5 receptors are key regulators of astrocyte activity. Synaptic release of glutamate activates mGluR5 expressed in perisynaptic astrocytes and generates intense Ca^2+^ signaling in these cells. Ca^2+^ oscillations, in turn, trigger the release from astrocytes of the gliotransmitter glutamate, which modulates neuronal excitability by activating NMDA receptors. By integrating these results with the most recent evidence demonstrating the importance of astrocytes in the regulation of neuronal excitability, we propose that astrocytes are involved in mediating some of the mGluR5-dependent drug-induced behaviors.

## INTRODUCTION

During the past 20 years, there has been a revolution in our understanding of the active roles of astrocytes. Although we initially knew that they played pivotal roles in performing metabolic functions, in taking up elevated $K^+$, and in clearing synaptic transmitters, we now appreciate that astrocytes can release chemical transmitters through a process called gliotransmission, which modulates neighboring neurons[1,2]. At this point, we therefore understand that astrocytes are active signaling elements in the nervous system, although we do not yet appreciate their roles in neuronal network function, behavior, or whether alterations in the basic biology of the astrocyte and gliotransmission could lead to long-term changes in nervous system function.

Our goal is to review some of the basic biological breakthroughs that have led to our new appreciation of glial function, and then integrate this knowledge into potential functions in reward centers of the brain to illuminate how glia might play important roles in responding to drugs of abuse, including cocaine and alcohol. With the development of molecular genetic tools to perturb the astrocyte selectively, the stage is now set for future studies to determine whether and how astrocytes contribute to both the function and dysfunction of the nervous system.

## ASTROCYTES ARE EXCITABLE

Electrical recordings from astrocytes show that they have a very negative resting potential, low input resistance, high resting conductance that is selective for $K^+$, and they are electrically unexcitable. Consequently, little attention has been focused on these cells during the past century, given that it was determined early in the 20th century that electrical excitability is a fundamental property of the nervous system that is essential for mediation of behavior. However, in the early 1990s, the development of ion-selective indicators and advanced forms of microscopy allowed a re-examination of astrocyte excitability. Initially in cell culture[3,4], and later *in situ*[5,6] and *in vivo*[7], $Ca^{2+}$ imaging demonstrated that astrocytes respond to the application of chemical transmitters with an elevation of their internal $Ca^{2+}$ levels. Given that astrocytes are not electrically excitable, it was rapidly realized that voltage-gated $Ca^{2+}$ conductances could not mediate $Ca^{2+}$ accumulations, rather that $IP_3$-gated release of $Ca^{2+}$ from internal stores was the prevalent source of $Ca^{2+}$ that mediated $Ca^{2+}$ oscillations observed in astrocytes[2]. More recent studies show that the stimulation of presynaptic axons in brain slice preparations evokes $Ca^{2+}$ oscillations in astrocytes, and that sensory stimulation *in vivo* is able to activate this form of excitability within these non-neuronal cells[8]. Typically spontaneous and activity-evoked $Ca^{2+}$ signals in astrocytes *in situ*[5,9,10] as well as *in vivo*[7] display an oscillatory pattern consisting of repetitive $Ca^{2+}$ spikes, which we will refer to in the rest of the manuscript as $Ca^{2+}$ oscillations.

A hallmark of neuronal function is the use of ligand-gated ion channels to cause rapid neuronal depolarizations in response to the release of synaptic transmitters. Though astrocytes can express such receptors, more typically it is the metabotropic receptors that respond to synaptic transmitters. For example, following stimulation of the Schaffer collaterals in the hippocampus, the transmitter that spills over from the synaptic cleft activates a combination of metabotropic glutamate and purinergic receptors to induce the release of $Ca^{2+}$ from $IP_3$-sensitive $Ca^{2+}$ stores within astrocytes[11]. Astrocytes express a plethora of metabotropic receptors[2] and, as will be discussed later, one of particular interest is metabotropic glutamate receptor 5 (mGluR5).

## GLIOTRANSMISSION: THE RELEASE OF CHEMICAL TRANSMITTERS FROM ASTROCYTES

After the initial observations of $Ca^{2+}$ excitability within astrocytes, it was not long until at least one of the consequences of these $Ca^{2+}$ signals was identified. As has been typical for this field, initial observations in cell cultures, then later in brain slice preparations, showed that a $Ca^{2+}$ elevation within the astrocyte was necessary and sufficient for the release of chemical transmitter from an astrocyte. The application of bradykinin, which mobilizes $Ca^{2+}$ from the internal stores of astrocytes, caused glutamate to be released from purified cultures of astrocytes[12]. This process of glutamate release critically requires elevations of internal $Ca^{2+}$ because chelating internal $Ca^{2+}$, by the addition of an exogenous $Ca^{2+}$ buffer, inhibited bradykinin-induced glutamate release. Furthermore, bypassing the receptor using photolysis of caged $Ca^{2+}$ also stimulates glutamate release[13,14,15].

In cell culture, our group had shown that astrocytic $Ca^{2+}$ oscillations induce delayed neuronal $Ca^{2+}$ elevations that are stimulated via glial glutamate release acting through NMDA receptors[16]. Subsequently, similar observations were made in acute brain slice preparations indicating that gliotransmission observed in culture is functional in relatively intact brain slice preparations[6,17].

Though imaging showed that astrocytes could signal to neurons, electrophysiology has been required to determine physiological roles for this pathway. Numerous laboratories demonstrated that astrocytes release glutamate and by activating neuronal receptors, these glia control synaptic transmission and neuronal excitability. Initial culture studies demonstrated that gliotransmission can modulate miniature EPSC frequency[18], as well as cause NMDA receptor-dependent slow inward currents (SICs)[19,20]. Later, these observations were confirmed in brain slice preparations[14,21,22,23,24,25, 26,27,28] and were shown to be mediated by a variety of glutamate receptors: kainate, mGluR, and

NMDA (Fig. 1)[15,29,30,31]. One difficulty associated with this type of study is to provide selective stimuli to the astrocyte in order to identify glial-evoked neuronal responses; since astrocytes are not electrically excitable, a nonelectrical stimulus is required. Photolysis has been the preferred strategy as it has been possible to provide absolute cell specificity[13,14,15,29]. Thus, in the hippocampus, photorelease of $Ca^{2+}$ or $IP_3$ within a single astrocyte has been shown to cause glutamate-mediated modulation of synaptic transmission as well as neuronal excitation. Additionally, to control for potential roles of presynaptic release of glutamate, studies have shown that the inhibition of synaptic transmission does not alter gliotransmission-mediated excitation of neurons[14,22,32]. Together these studies have provided unequivocal evidence for the presence of gliotransmission in the nervous system.

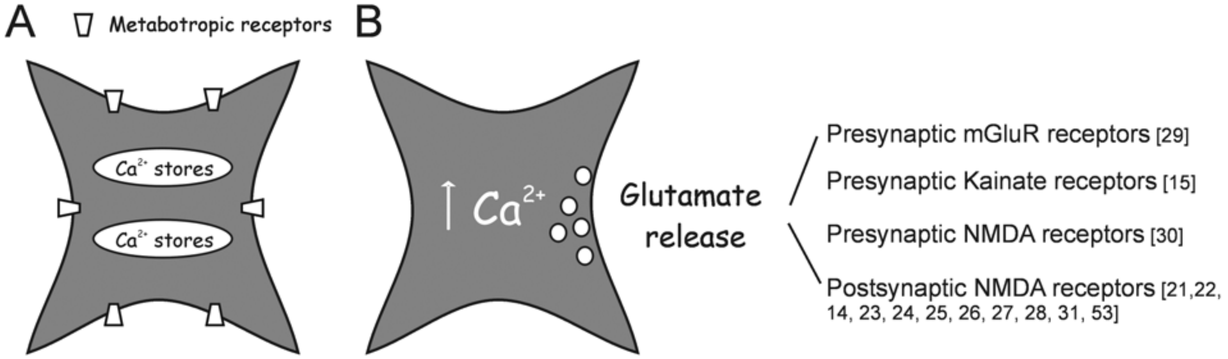

**FIGURE 1**. Glutamate release from astrocytes *in situ* controls neuronal excitability. (A) Astrocytes express metabotropic receptors for different neurotransmitters in the plasma membrane. Activation of these receptors triggers $Ca^{2+}$ increases (B) within astrocytes due, mainly, to $Ca^{2+}$ release from intracellular stores. (B) These $Ca^{2+}$ oscillations stimulate the release of glutamate from astrocytes, which modulates neuronal excitability through the activation of a variety of different neuronal receptors.

## DIFFERENTIAL CA$^{2+}$ CODES

Three recent studies have shown that it is inappropriate to think of astrocytic excitability simply as a $Ca^{2+}$ elevation. Unlike the action potential that we think to be all-or-none, $Ca^{2+}$ elevations in astrocytes come in many forms that can have differential actions. One form of transmitter release from the astrocyte is mediated through exocytosis[33]. Using total internal reflection microscopy to image fusion of vesicles with the astrocytic plasma membrane, Baljit Khakh's group has shown that two different ligands that cause $Ca^{2+}$ elevations of similar magnitude lead to different forms of exocytosis[34]. ADPβS, which activates purinergic receptors, causes short-duration $Ca^{2+}$ elevations (~5-sec duration) and a form of exocytosis consistent with kiss-and-run exocytosis, while thrombin, which activates PAR-1 receptor, leads to $Ca^{2+}$ elevations of ~80-sec duration and to full-fusion exocytosis. Similarly, Chen et al. showed that different stimuli applied to astrocytes in culture can differentially cause kiss-and-run and full-fusion exocytosis[35]. What about *in situ*? A recent study from McCarthy's group strongly supports the notion that the form of $Ca^{2+}$ elevation is essential for stimulating transmitter release[36]. They expressed a foreign receptor in astrocytes to allow selective pharmacological activation of astrocytes. Although activation of this receptor stimulated long-duration $Ca^{2+}$ plateaus in astrocytes, neuronal actions were not detected, a result that is in disagreement with the expectations set by the work of numerous laboratories including their own[14,21,22,23,24,29]. However, when they photoreleased $IP_3$, which causes transient, short-lasting $Ca^{2+}$ increases, they were able to evoke glutamate release from the glial cell that was detected as a modulation of synaptic transmission. There are several potential explanations for this disparity, which include the possibility that the receptor is not present in the fine glial processes responsible for transmitter release. However, a more exciting possibility is that the different forms of $Ca^{2+}$ signal evoked by these two activation pathways lead to differential effects: ligand-induced $Ca^{2+}$ elevations were plateau events lasting for 200–300 sec, while $IP_3$-induced elevations were of the order of 10–20 sec in duration. Perhaps relatively brief oscillations are required for evoking transmitter release. When taken together with the observation that some receptors, such as endothelin-1, which induces $Ca^{2+}$ oscillations, can also inhibit exocytosis through parallel signaling pathways[37,38], it is clear we have entered into a new era where it is critical to decode the pathways activated by receptors in order to appreciate the richness of signaling possibilities within the astrocyte.

## A DIVERSITY OF GLIOTRANSMITTERS

In addition to glutamate, we now know that astrocytes release additional transmitters that can have neuronal actions[39,40]. Astrocytes release ATP, which can have direct neuronal actions. Additionally, once released into the extracellular space, ectonucleotidases hydrolyse ATP to adenosine, which inhibits neurons by suppressing synaptic transmission[41] and by activating $K^+$ channels[42].

Serine racemase, an enzyme that can convert L-serine to D-serine, is expressed by astrocytes and is responsible for the accumulation of D-serine within these glial cells[43]. D-serine, which is released from astrocytes, is a coagonist for the NMDA receptor binding to the so-called glycine-binding site. Selective degradation of D-serine (but not glycine) by D-amino acid oxidase (DAAO) markedly reduced the NMDA-dependent transmission in the hippocampus[44]. This result was then replicated in other brain areas[45], confirming the hypothesis that astrocytes contribute actively to NMDAR-dependent processes in the mammalian brain through the release of the gliotransmitter D-serine. Thus, astrocytes have the potential to regulate neuronal function through several transmitters. At this time, we do not know whether glutamate, D-serine, and ATP are released from the same astrocyte, or whether by analogy with neurons, there are distinct purinergic, glutamatergic, and D-serinergic astrocytes.

## GLIOTRANSMISSION IS REQUIRED FOR ADENOSINE-MEDIATED SUPPRESSION IN THE HIPPOCAMPUS

So far, we have only discussed the examples where studies have shown that astrocytes and gliotransmission are able to induce neuronal responses. Whether these pathways are required by the nervous system for brain function has been more difficult to determine. Molecular genetics has been applied to the astrocyte to inhibit purinergic gliotransmission and revealed a critical role of astrocytes in the control of extracellular adenosine[41]. The conditional expression of a dominant-negative SNARE domain (dnSNARE) selectively within astrocytes caused an enhancement of synaptic transmission within the hippocampus. It has been known for many years that there is a tonic level of extracellular adenosine in the nervous system that causes a presynaptic inhibition of synaptic transmission mediated by A1 receptors[46]. When the dnSNARE was conditionally expressed in astrocytes, this adenosine-mediated inhibition was absent[41].

In addition to demonstrating an essential role for the astrocyte in the control of synaptic transmission, these studies showed that adenosine is controlled by the astrocyte. Alcohol induces the accumulation of adenosine by inhibiting type 1 equilabrative transporters that are normally responsible for taking up extracellular adenosine[47]. Since our hippocampal studies demonstrate an important role for the gliotransmission controlling extracellular adenosine, it is tempting to speculate that astrocytes play important functions in responses to alcohol and potentially to alcohol addiction. The availability of the astrocyte-specific dnSNARE mouse should allow these studies to be performed.

## mGLUR5 STIMULATES GLIAL EXCITABILITY AND GLIOTRANSMISSION IN THE NUCLEUS ACCUMBENS

Class I metabotropic receptors are known to play important roles in evoking $Ca^{2+}$ signals in astrocytes in response to synaptic activity[5]. Additionally, one of these receptors, mGluR5, is known to play essential roles in mediating some of the actions of drugs of abuse. mGluR5 knockout mice do not exhibit cocaine self-administration behavior[48] and mGluR5 antagonists can prevent cocaine, nicotine, and morphine drug-seeking behavior[49,50,51,52]. Because of the importance of mGluR5 in mediating these behaviors and because of its importance in the activation of astrocytic $Ca^{2+}$ signaling, we have recently performed a study on the consequences of astrocytic mGluR5 activation in the core of the nucleus accumbens, a key region for drug-seeking behavior[53].

Immunocytochemistry demonstrates that mGluR5 colocalizes with astrocytes (Fig. 2A–C) in the nucleus accumbens and that addition of an mGluR5 agonist induces astrocytic $Ca^{2+}$ oscillations in this nucleus[53]. This raises the potential that spillover of transmitter from glutamatergic synapses could induce astrocytic signaling. Indeed, stimulation of glutamatergic afferent pathways induces astrocytic $Ca^{2+}$ oscillations that are attenuated by the mGluR5 antagonist, MPEP (Fig. 2D,E). Because MPEP does not affect synaptic transmission at the low concentration we used (10–20 $\mu M$), these results support the idea that synaptic activity can activate astrocytic $Ca^{2+}$ signals by engaging mGluR5 within these glial cells.

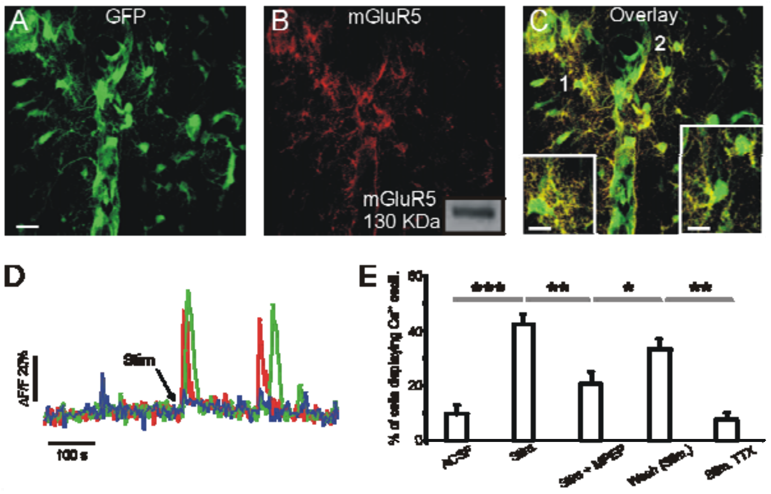

**FIGURE 2**. mGluR5 receptors mediate astrocyte activation following stimulation of neuronal afferents. (A–C) mGluR5 receptors (B, red) are expressed in nucleus accumbens astrocytes, identified by the expression of GFP (A, green). (D,E) Stimulation of glutamatergic afferents to the nucleus accumbens with trains of stimuli results in mGluR5-dependent activation of $Ca^{2+}$ signaling into astrocytes. $Ca^{2+}$ signals consist of repetitive, brief $Ca^{2+}$ spikes. (Modified from D'Ascenzo et al.[53], copyright *Proc. Natl. Acad. Sci. U. S. A.*)

We then have asked whether astrocytic $Ca^{2+}$ signaling leads to gliotransmission in the nucleus accumbens as has been shown elsewhere in the nervous system. Photorelease of caged $Ca^{2+}$ within astrocytes causes SICs in medium spiny neurons (Fig. 3A–C), which are attenuated by the NMDA receptor antagonist D-AP5[53]. Moreover, synaptic activation also recruits this pathway of gliotransmission. Synaptic stimulation causes astrocyte-dependent neuronal excitation in the form of SICs that can be detected for minutes following only seconds of synaptic activity (Fig. 3D,E). Moreover, in agreement with the $Ca^{2+}$ imaging data, this pathway requires activation of mGluR5 (Fig. 3F). Taken

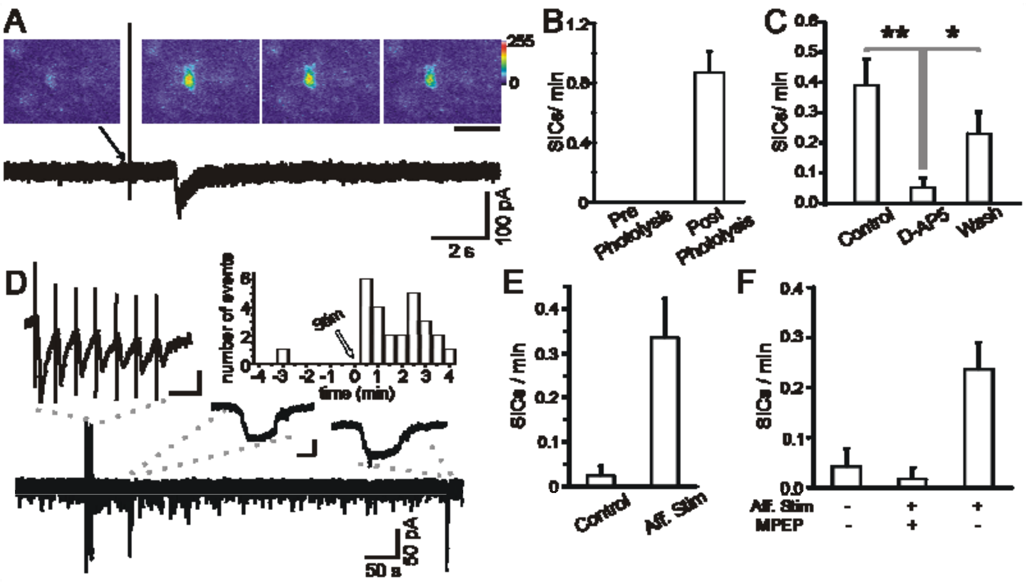

**FIGURE 3**. $Ca^{2+}$-dependent glutamate release from astrocytes generates SICs in medium spiny neurons. (A–C) Selective photolysis of astrocytic caged $Ca^{2+}$ results in the detection of SICs in nearby nucleus accumbens neurons. (D–F) Glutamatergic afferent stimulation activates astrocytic $Ca^{2+}$ (see Fig. 2), activates $Ca^{2+}$-dependent gliotransmission, and thus increases the frequency of SICs recorded in medium spiny neurons. Activation of astrocytes and, consequently, the detection of SICs are blocked by the mGluR5 antagonist MPEP (F). (Modified from D'Ascenzo et al.[53], copyright *Proc. Natl. Acad. Sci. U. S. A.*)

together, these studies show that glutamatergic synaptic activity in the nucleus accumbens leads to mGluR5-dependent release of $Ca^{2+}$ from astrocytic $IP_3$-sensitive stores, which induces the release of glutamate that then causes a feed-forward excitation of medium spiny neurons that is mediated through NMDA receptors (Fig. 4).

A particularly interesting feature of synaptic activation of astrocytes is that glial excitability is prolonged. Brief stimulation of afferents leads to oscillations that last for hundreds of seconds (Fig. 2D). Because these $Ca^{2+}$ oscillations induce gliotransmission, which causes a feed-forward excitation of medium spiny neurons (Fig. 4), an intriguing possibility is that even relatively brief exposure to drugs of abuse will cause an mGluR5-dependent excitation of astrocytes that will cause prolonged activation of neurons.

## SUMMARY AND PERSPECTIVES

Although understanding how drugs of abuse produce long-lasting neuroadaptation that underlies drug-seeking behavior and relapse is one of the fundamental issues in neuropsychology, there has been little investigation of the potential role of astrocytes in mediating these processes. However, three observations suggest that attention should be turned to these non-neuronal cells. First, Kalivas' group has shown an important role for cystine-glutamate transporters, which are thought to be glial transporters, in preventing reinstatement of drug-seeking behavior[54]. Second, mGluR5, which is essential for mediating certain behavioral responses of drugs of abuse, activates gliotransmission[53]. Finally, Bowers and Kalivas[55] showed an increase of GFAP expression in an animal model of cocaine addiction (a process termed reactive astrocytosis). This process can involve changes in gene expression and in the physiological properties of these glia cells, including $Ca^{2+}$ excitability and gliotransmission. Given that these changes are prolonged (potentially lifelong), it will be of importance to direct experimental studies to these cells to ask whether they mediate some of the effects of drugs of abuse in the nervous system.

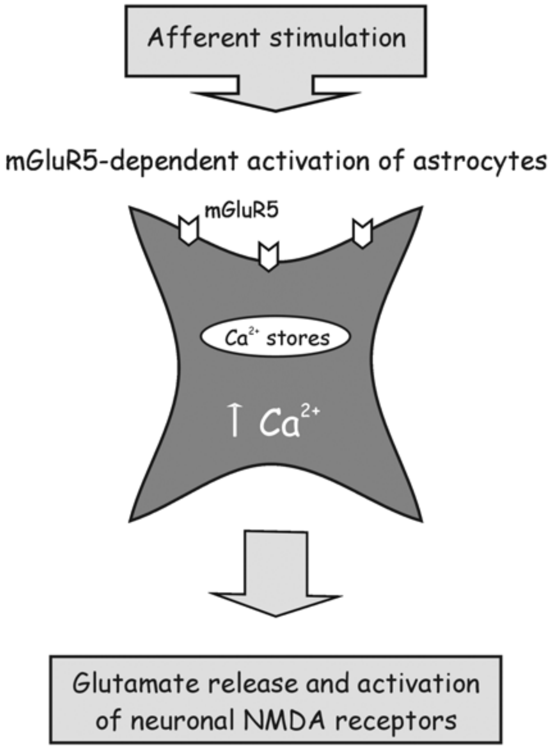

**FIGURE 4**. mGluR5 receptors are key regulators of gliotransmission in the nucleus accumbens. Model depicting the sequence of steps leading to $Ca^{2+}$-dependent gliotransmission. Spillover of glutamate following glutamatergic afferent stimulation results in the activation of astrocytic mGluR5 receptors, which generate sustained $Ca^{2+}$ oscillations into astrocytes. The oscillatory $Ca^{2+}$ signals trigger the release of glutamate from these cells, which activates neuronal NMDA receptors.

## ACKNOWLEDGMENTS

The authors' work is supported by the NINDS and NIMH.
